# Supplementary material for: Narrative event segmentation in the cortical reservoir
Source: PLoS Comput Biol. 2021 Oct 7;17(10):e1008993. doi: 10.1371/journal.pcbi.1008993 (PMC8525778; doi:10.1371/journal.pcbi.1008993)
Supplement: Attachment — Submitted filename: PLoSCB-reviews.pdf [file pcbi.1008993.s001.pdf]

Dear Editor and Reviewers,

First let me thank you and say that it is a real pleasure to see such reviews and editorial remarks that show a clear reading of the paper, and comments that are insightful and provide the basis for improving the paper. I have addressed all the comments, as indicated in detail below. I hope you find the revision appropriate for publication.

Cheers

Peter Ford Dominey

Reviewer 1:

Major comments:

R1.1 As someone who's not very familiar with this kind of network model, I think the manuscript would benefit from a bit more exposition early on about how the reservoir network works. We get some details in the Discussion and the Methods section located at the end of the paper, and at some points the author points to a previous paper (Uchida et al., 2021); but

- there should be sufficient "standalone" details about the reservoir network in this paper, and these should come early enough in the Introduction/Results that the reader can more easily interpret the findings.

  * Thanks for pointing this out. I am too "reservoir-centric"! I now include a more complete and standalone description of the reservoir in the introduction so as to address all these questions on pages 3 and 4.

- For example, you say "the reservoir was trained to generate the discourse vector"—but how exactly? What's the training objective?

  * I now specify that the training objective is to learn the vector average of the input embeddings.

- Is the reservoir network previously trained on the text corpus somehow? You mention in the Discussion that in the "current modeling effort we do not train the reservoir," but this comes very late (and is still a bit opaque to me).

  * I now clarify that while there is no learning in the reservoir, the reservoir receives as input the Wikipedia2Vec embeddings which benefit from the massive training of the Wikipedia2Vec model.

- Another example: How do the temporal dynamics (time constants) of the network arise? Do they emerge out of some sort of training, or are they user-specified parameter settings when wiring the reservoir? (Apologies if these things are obvious and I'm just missing it!)
-

* This is now explained in the introduction.

R1.2 The choice of applying event segmentation to downsampled whole-brain fMRI data doesn't seem to have a strong precedent in the related literature. For example, Baldassano et al., 2017, and 2018, use searchlights and/or focus on high-level association areas like angular gyrus, posterior medial cortex, and medial prefrontal cortex. In this case, putative event representations are encoded in finer-grained response patterns within a given region of interest (although not too fine-grained via Chen et al., 2017). It's not clear to me that these sort of response patterns seen in the literature contribute meaningfully to the coarse, whole-brain response patterns used here, and I'm somewhat surprised the event structure is evident at the whole-brain level (e.g. Fig. 4A). This non-localized approach also seems to be in tension with the idea of a cortical hierarchy with different temporal dynamics. Replicating this analysis with more localized cortical areas implicated in high-level event representation could strengthen the argument; otherwise, better motivation should be provided for using coarse whole-brain data.

* Indeed. We now perform this experiment using data from the angular gyrus (AG) while subjects watched the first 24 minutes of an episode of Sherlock (data from Chen 2017, provided by Baldassano) and the transcript from recall of that same video segment (data from Zadbood et al (2017)). This is updated in the manuscript.

R1.3a There are several places where the choice of the predefined number of events k for the HMM seems arbitrary or insufficiently motivated. For example, why is a HMM with k = 5 used when applying the reservoir network to the Wikipedia-based test narrative generated from four Wikipedia articles? Were multiple values of k assessed (which values?) and k = 5 chosen (based on what criterion?)? Or did you try k = 4, but the result fit poorly?

* In each case we now justify choices of k, and when appropriate we do exhaustive search on k. In the segmentation of the Wikipedia and NYT texts, we have now combined them into a single longer text made up of 8 segments. Starting at k=8 we run the HMM and increase k until the 8 segments are identified. This yields k=10.

R1.3b Other examples: when running the HMM on the fMRI data, you specify k = 10; when you examine the fast and slow reservoir subnetworks, you use k = 25 and k = 8—why? If you're trying multiple values of k here, it should be a systematic comparison and we need to know the criteria for selecting k; for example, I would consider using the t-distance introduced by Geerligs et al., 2021; there's also a nice demo here: https://naturalistic-data.org/content/Event_Segmentation.html.

* In this section comparing segmentation for angular gyrus and the model, we chose k based on data from Baldassano that indicates suitable values of k for different cortical areas. We state:

> Baldassano et al (2017) determined that the optimal segmentation granularity for AG is between 50-90 segments for the 1976 TRs in the 50 minute, corresponding to a range of 24-43 segments for the 946 TRs in the 24 minute fMRI data we used. We thus chose k = 40 for the HMM segmentation, as a value that has been established to be in the optimal range for AG.

* In the section comparing the segmentation of representations in the fast and slow virtual areas, we now perform an extensive analysis of the k values. We first determined k values for the fast and slow that were chosen based on values reported in Baldassano for fast visual areas, and slower associate areas, and we also performed an exhaustive search on k values from 2 to 40, using the log-likelihood of model fit.

This is presented in the results, and the methods are described in a specific new paragraph in the methods section. We discuss Geerligs method in the discussion.

R1.4 I'm having a hard time understanding how event boundaries are statistically compared here. For example, you report for the reservoir network that the "ground truth and HMM segment boundaries are highly correlated, with the Pearson correlation $r = 0.99$, $p < 0.0001$." What exactly is being correlated here? Are you correlating a time series of zeros with ones where an event boundary is found? In this case, the degrees of freedom is the number of (autocorrelated) time points. I'm not sure this sort of statistical test is adequate and would advocate for a nonparametric randomization-based approach. For example, Baldassano et al., 2017, use a randomization procedure where they shuffle the boundaries (e.g. 1000 times) while preserving the duration of the events (in conjunction with some metric like the t-distance mentioned above).

* We now use the shuffling method of Baldassano to assess the fit between segmentation of NIR and fMRI as you suggest – thanks! This is described in detail in a new paragraph on p. 9.

R1.4b In line with the previous comment, when comparing the boundaries (at $k = 5$) found for the NYT and Wikipedia test narratives, you say you "normalized the resulting event boundaries into a common range, and made a pairwise comparison of the segment boundaries for the two texts." I'm not really sure what this means. You compared the index of the time points on which each of the four boundaries landed? But your t-value has 5 degrees of freedom, suggesting 6 boundaries were compared… including the first and final time point? Again, I think a nonparametric statistical approach for comparing segmentations (e.g. adapted from Baldassano et al., 2017) would make this more convincing.

* Again, we have replaced this comparison with the Baldassano method, and we also compare the reservoir to the embeddings and a linear integrator – see next comment and response.

R1.4c In Figure 11, you show the pre-reservoir time-point correlation matrix for Wikipedia2Vec embeddings that serve as input to the network. The lack of slow, event-like structure seems obvious here, but it could be useful to treat this as a more formal "control" model. In other words, if you want to show that the reservoir network captures narrative structure above and beyond the word-level embeddings, it might be worthwhile to show that it provides statistically better event segmentations than the pre-reservoir embeddings.

* Good suggestion! A similar point was raised by Reviewer 2. We examine HMM segmentation on the embeddings alone and on a linear integrator, and show event structure in the embeddings, and in the linear integrator mode. We measure their performance vs. ground truth using the method you suggest from Baldassano. The temporal processing of the reservoir beyond the embeddings and linear integrator is now described in the text, and also mentioned in the abstract.

R1.5 This paper demonstrates that relatively straightforward recurrent dynamics can reproduce several of the temporal dynamics observed in fMRI data during narrative comprehension. However, the modeling work here doesn't really touch on the actual content of those high-level event or narrative representations. For example, Baldassano and colleagues relate event representations to situation models. Do we have any interpretation of the discourse vectors represented by the reservoir network (other than summarizing the prior semantic vectors)? You touch on this in the Discussion on page 16, but it might deserve an additional sentence or two.

* I have now added a new paragraph addressing meaning in more detail in the last two paragraphs of the discussion p. 20-21..

The figures are described in the main text, but I don't see any figure captions in the copy of the manuscript provided by the journal or on bioRxiv. Standalone figure captions would be helpful.

* that has been addressed

Minor comments:

For figures with time-point similarity matrices (e.g. Figs. 2, 4), it would be helpful to see color bars so we have an intuition about the scale of correlations(?) observed.

* Done. Color bars are now on all graphs where appropriate.

Abstract: not sure you need "awake" in the first line here

* deleted

Abstract: expand "HMM" acronym

* done

Page 4: "Wikipedia2ec" > "Wikipedia2Vec"

* done

Page 5: "has to with" > "has to do with"

* done

Page 7: "Braniak" > "BrainIAK"

* done

Figure 7: "Alignement" > "alignment"

*done

Page 8: I would use the full story name and credit the storyteller here: “It’s Not the Fall that Gets You” by Andy Christie (https://themoth.org/stories/its-not-the-fall-that-gets-you)

* we now cite this as suggested

Page 9: You note the 10 events assigned to the fMRI data and say “Likewise for the Narrative Integration Reservoir, 10 instances were created…” This wording implies to me that the number of reservoir instances relates to the number of events—but I think you want to say that the number of instances is matched to the number of subjects (also 10). Also, you say you “summary results of this segmentation”—but how do you summarize across instances?

* This is clarified. 16 reservoirs are used to have the same number of instances as the number of fMRI files. We also now state the we take a mean of the set of boundaries determined for the multiple reservoir instances..

Page 14: “remain” > “remaining”

* OK

Page 15 “reservoir correlations displayed an abrupt response in the forgetting context” due to “immediate responses to input in the reservoir”—this still wasn’t very intuitive to me… why?

* This effect is due to the leaky integrator property of the reservoir. Indeed, we see similar behavior with the linear integrator. We now illustrate this in Figure 5.

Great to see the code on GitHub!

* Thanks!

References:

Baldassano, C., Hasson, U., & Norman, K. A. (2018). Representation of real-world event schemas during narrative perception. Journal of Neuroscience, 38(45), 9689-9699. https://doi.org/10.1523/JNEUROSCI.0251-18.2018

Chen, J., Leong, Y. C., Honey, C. J., Yong, C. H., Norman, K. A., & Hasson, U. (2017). Shared memories reveal shared structure in neural activity across individuals. Nature Neuroscience, 20(1), 115-125. https://doi.org/10.1038/nn.4450

Geerligs, L., van Gerven, M., & Güçlü, U. (2021). Detecting neural state transitions underlying event segmentation. NeuroImage, 236, 118085. https://doi.org/10.1016/j.neuroimage.2021.118085

Reviewer #2:

MAIN POINTS

R2.1 The most important missing piece in this manuscript is to present a comparison model

that does /not/ produce the effects exhibited by the narrative reservoir model. In particular, it is crucial to demonstrate that the model's performance is different from something very simple like feeing the embeddings into a linear integrator model, or a linear filter [e.g. a boxcar running average, or a recency-weighted average like [0.2, 0.18, 0.16, 0.14, 0.12, 0.08, 0.06, 0.04, 0.02] . More generally, it would be fascinating to know which of the elements of the reservoir model are necessary in order to account for the effect: for example, what happens if leak rate is set to near-zero, or if it is set to a very high value? Together such comparisons could help to understand whether nonlinearity is even required to generate the effects, and to separate out which effects arise from the reservoir component, and which effects arise from the wikipedia2vec embedding model.

* Great point. We now present results from a comparison with a linear integrator model. This model indeed produces a very similar integration effect, including the fast forgetting and slow construction. Importantly it does not yield the distribution of time different timescales, and thus cannot replicate the hierarchical pattern of event segmentation, context construction and context forgetting.

The linear integrator is described in the methods section, and the results of the experiments with embeddings, linear integrator with different time constants, and reservoir with different time constants are now presented in the first section of the results.

This indeed makes a significant improvement, because we see that what the reservoir provides is the diversity of temporal structure representation.

R2.2 Related to the point above, the manuscript claims that the results do not require any learning. For example, in this paragraph: "It is worth noting, in the context of the relation between narrative structure and recurrent network functional neuroanatomy, that the observed reservoir behavior comes directly from the input-driven dynamics of the reservoir. There is no learning, and we do not look at the readout, but at the recurrent elements of the reservoir itself."
Although I understand that the reservoir model is not trained in any way to match the neural data, the model does have the benefit of the enormous amount of information (learned from text and semantic-structure) in the encoding model. At a conceptual level, please textually clarify the sense in which there is no learning. At a more practical level, it seems critical to better characterize how much event-structure can be directly extracted from the embeddings themselves. Figure 11 shows a comparison of the word-embedding autocorrelation structure and the reservoir-state autocorrelation structure, and these do appear to be very different. However, they are plotted on different scales, and there is no smoothing at all applied to the word-embeddings. It seems implausible that words sampled from within one Wikipedia article (or New York Time article) are going to have the same average inter-word similarity as words samples across two distinct articles — certainly many words will be shared or non-specific, but in the time-averaged data, there should be some semantic "themes" that are shared across sentences within an article, but not across articles. It is crucial to separate out such effects [inherent in the input to the narrative reservoir model] from the effects that arise from the recurrent dynamics of the reservoir model.

* Thanks – this is an important point that was not clear. This part of the discussion is now updated to take into consideration the information already in the embeddings, including discussion of the new experiments directly using the embeddings, and a linear integrator

model.   We clarify what "no learning" means (page 19) – including that while there was significant learning in the Wikipedia2Vec model which the reservoir benefits from, there was no learning in the reservoir itself. We also identify the specific added value of the reservoir.

This point is also clarified with the new analysis of embeddings and linear integrator, showing that they indeed have information, which is due to the extensive learning in the Wikipedia2Vec model.

R2.3 The HMM segmentation model will probably have a bias to "equally space" its events across an interval from start to end — in other words, in the absence of any actual structure over time, such as in random noise data, the HMM will likely segment a sequence into units of similar length. Therefore, in order to compare segmentations in the neural data and in the model, it seems important to run a control in which we use the HMM to cluster a /permutation/ of the real data, and then show that the HMM fit on this permuted data has a lower correspondence to our model [or fMRI data] than the HMM fit on the original. For example, if we have a sequence of events ABCD in the simulation and the same sequence ABCD in the neural data, then we cannot just show that the timepoints of the segmentations are correlated — the more compelling demonstration would be [for example] to compare (i) correlation when simulation and neural data are both using ABCD ordering and (ii) correlation when the simulation uses DBAC ordering and the neural data is for ABCD ordering.

* Thanks. This is an important point. We now address this issue, using the permutation method of Baldassano (2017) to assess the fit between segmentation of NIR and fMRI. This is described on page 11. We also use this permutation method in assessing the NIR model against ground truth in the corpus we made from New Your Times and Wikipedia, described on page 9.

R2.4 There are some issues relating the timing of the words in the (auditory, spoken stimulus, for which the neural data were recorded) and the location of words within the text-transcript fed into the word embedding model. Words are not spoken at a constant rate in a narrative, to 50% of the words do not correspond precisely to 50% of the time in a narrative. In order to align neural data (fMRI timing) with model predictions (word-embedding timing), the only solution is to determine when each word is spoken. The authors propose two methods for aligning the neural data with the stimulus timing, but (as far as I can tell?) neither of these methods actually precisely aligns the timing of the neural data with the timing of when the actual words were spoken. Since the stimuli are all available, is it not possible to generate the simulations in a way that matches directly with when the words were spoken (and when the brain responses were recorded)?

* Thanks for this comment. We now note that the study of (Baldassano et al 2017) showed that the HMM can be used to find common event structure in neural data from different modalities with different timing, and that it is not necessary to have a precise temporal alignment between them. We now base our experiment on data from this experiment, and we now explain this in the beginning of the section on "Comparison of Narrative Integration Reservoir and Human fMRI Segmentation on corresponding input" (page 24).

R2.5 When illustrating the time-time correlation maps, it may be helpful to do so only after subtracting the mean value from each element (i.e., for each unit in the reservoir, or each dimension in the word-embedding, treat the signal as a time-series, and subtract the mean of the time-series from every time-point). This can prevent saturation issues which may confuse the interpretation. For example, in Figure 11 there is an illustration of the time-time correlation for the embedding inputs (panel B) and for the reservoir states (panel D). One correlation map is shown on a scale from 0.0 to 1.0 and the other maps is shown on a scale from 0.88 to 1.0. If the mean values are removed from all elements, then the maps will not have these saturation effects, and they can plotted on comparable scales. The saturation effect (where all correlation values are very high) arises when there is a common “mean signal” that is stable within a system over time, so that all pairs of timepoints are highly correlated.

* Thanks very much for this. This significantly improves the visibility of the results in the figures. We now use this method and mention this in the methods section.

R2.6 The forgetting curves, grouped by timescales, shown in Figure 9, exhibit a dip at the beginning and then a spike around t =15. It is not entirely clear what is happening at t=0 and what is happening at t=15. Please could you make this clearer, both in the text on the figure using labels. Given that the model is driven by word embedding, is it possible that sudden “separation” or “convergence” effects arise from high/low frequency characters (e.g., special characters such as punctuation, or onset-related words) that occur at the event boundary? It is important to rule out the possibility that the model is driven to an unusual state by distinctive words or characters that occur near event boundaries, rather than by the broader “meaning incompatibility” between the prior context and the current input. Does this sudden dip (Figure 9) occur for all event boundaries and/or for all sentence boundaries? [Of course, it does seem that the patterns of the units were driven by the stimulus after the event boundary, and the curves seem to separate over time indicating their representation are gradually different.]

* We now clarify this. We add two panels to the figure to clearly illustrate what is happening. We recall that we have two reservoirs, one receiving the intact narrative ABCD and the other receiving a scrambled narrative ACBD (where A-D are continuous subsets of words making up the “Not the fall” text). Figure 9 illustrates the difference in the activity in the two reservoirs. During the common narrative segment A, the difference is null. This corresponds to the activation difference of zero in panel C (of the new figure) and the flat line at 0 in panel A. The dotted vertical line indicates the transition from A to B and A to C. At that point, the activation difference quickly diverges from 0 in panel A, and the correlation of 1 quickly diminishes in panel B. It reaches a minimum about 15 time steps later and then fluctuates around the same level.

This is updated and clarified in the figure caption and text (page 15).

R2.7 It was unclear to me whether this manuscript is proposing that reservoir dynamics are actually proposed as a process model for cortical dynamics in the human brain. If so, please clarify what are the architectural features that are being proposed -- what are the essential features of the reservoir that we should interpret as functional principles for the cerebral cortex?

* The recurrent dynamics provide two key elements of cortical function. The first, which we have already examined, is the projection of the inputs into a high dimensional space, which provides the universal computing characteristic characterized by (Maass et al 2002), and revealed by mixed selectivity in the neuronal coding (Enel et al 2016, Rigotti et al 2013). The second property, which we investigate here, is the diversity of functional time constants that is a correlate of the high dimensional projection. Indeed, in this high dimensional representation, time is one of the dimensions (Dominey 1998).

This is specified in the discussion on p 19-20.

R2.8 I was intrigued by this section in the manuscript: " We can propose that this segregation is a natural product of neuroanatomy, and that narrative structure has found its way into this structure. Narrative structure reflects the neuroanatomy." Please could you extend and/or clarify this statement. As I understand it, the claim is that (i) each different stage of cortical processing could employ its own reservoir network and (ii) time constants in the reservoirs would be longer in higher order regions, and then (iii) this configuration would explain the results of Baldassano et al who analyzed narrative structure at multiple scales. If this is indeed the logic, please could you unpack this for the reader.

* Thanks for this opportunity: We now explain in the discussion top of page 19: While individual neurons have the same time constants, we observe a recurrent time constant effect, a functional variety of time constants due to the recurrent dynamics. We propose that in the primate brain, as higher cortical areas become progressively removed from sensory inputs, and driven by lower cortical areas, they will have higher effective time constants, due to the accumulation of the recurrent time constant effect, leading to the temporal processing hierarchy (Baldassano et al 2017, Chien & Honey 2020, Hasson et al 2008).

MINOR POINTS

In relation to the final "generalization" analysis, examining the long-vs-short scale event segmentation generalization, please provide a little more information about the generalization accuracy (beyond the difference between coherent vs. incoherent condition). For example, what is the generalization accuracy of event segmentation in each condition? Is there an accuracy difference between long vs. short timescale "regions"? Were meaningful events being segmented in the short- vs. long-timescale "regions"? Providing this information could help validate this analysis and clarify its meaning. Relatedly, the logic and procedure for the HMM-generalization analysis could be clarified in the text. The hypothesis being tested is described as follows: ""We can now test the hypothesis that there is a relation between the time constant for construction, and the granularity of segmentation in the Narrative Integration Model by the HMM." I think it may be clearer if you phrase this without reference to the HMM, since the HMM is just a data-analysis tool [it is not a process model for brain dynamics] — so I think that the underlying claim here is that differences in timescales of different units (within a reservoir model) can explain the increasing-scale event segmentation effects reported by Baldassano et al?

* Thanks for these questions. In response we have significantly reworked and simplified this analysis. In this experiment, we now directly compare segmentation over a range of k values for a fast and a slow virtual area. We present the segmentation results for these two areas over the range of k values. We clarify the statement of the hypothesis as suggested.

Figures: Please add axis labels to all panels of all figures, and ensure that the resolution is high; some figures are difficult to read (at least in the format supplied to reviewers)

* done

In Figure 4B, what is the low inter-event correlation (blue cross-shape) that happens in the middle of the story? Is there something very different happening over this period?

* We now use the Sherlock rather than the Not the Fall story, and this blue cross is not present.

Figure 5 nicely shows the effect of context forgetting and construction on the model representation. However, I find it hard to tell whether different units really “forget” the context at a similar rate. The forgetting curves and pattern correlation seem to take ~20 TRs to reach different activation/low pattern correlation. Is it possible to make this differentiation clearer or more explicit? Furthermore, although the color scheme allows to differentiate the correlation values, all the values are very high (above 0.95). This makes it harder to compare with the neuroimaging results in Chien and Honey (2020) where they subtracted from each voxel its mean signal.

* The forgetting process is now more clearly shown with more detail in Figure 6, and in Figure 9 we show that for the 5 different sets of neurons ordered by their construction rate, the forgetting curves overlap, showing that the forgetting occurs at the same rate. We make this more explicit in the explanation of Figure 9 on p 17.

Figures 5 and 6: The figure titles, captions and body text could be clearer on providing a summary “take home message” of these Figures.

* we now state at page 16 - The take home message of Figures 5 and 6 is that in the forgetting produces a rapid divergence between the reservoirs’ activations, while construction of a common context takes place over a much more duration.

Figure 6: In order to demonstrate that construction time and forgetting time are not related, would it not be more direct to plot forgetting-time vs construction-time unit-by-unit?

* The dissociation between construction and forgetting time is now clarified in Figure 9, where the forgetting rates for the 5 virtual areas are all the same, vs. the distribution of constructing rates.

Figure 6: there is no legend label for panels C and D

* corrected

Figures 8 and 9: I appreciated the finding that units could be grouped into different timescales based on context construction analysis. Given that the mapped timescale only ranges from 0-35, consider using just 3 groups to make the inter-group difference clearer. I did not perceive much difference between the 5 correlation maps shown in Figure 8, for example.

* For 8 we now show only two groups as you suggest, which makes the difference more visible. For 9, we prefer to keep the 5 separate areas to illustrate the continuous variation from fast to slow.

Figures 9-13: y-axis labels should state “activation difference” or similar (instead of “activation”)

* Thanks – this is the case for Figures 5, 6, 8 and 9 and the change has been made.

For the HMM hyperparameters, why was K = 5 chosen for Wikipedia, which also has 4 events as in the NY TImes text?

* These two texts have been combined now and the choice of k is explained.

Please elaborate on the initialization parameters for the narrative integration reservoir. What is the distribution of values from which W_in and W are randomly initialized? Does this choice powerfully shape the behavior of the reservoir, or would similar results be observed with any other choice, as long as the reservoir dynamics are neither diverging nor collapsing?

* We now elaborate that the W and W_in matrices are initialized with uniform distribution of values from -0.5 to 0.5. The leak rate is 0.05. We also tested with values0.1, 0.15 and 0.2. The reservoir is relatively robust to changes in these values, as long as the reservoir dynamics are neither diverging nor collapsing- on page 23.

Baldassano C, Chen J, Zadbood A, Pillow JW, Hasson U, Norman KA. 2017. Discovering event structure in continuous narrative perception and memory. *Neuron* 95: 709-21. e5

Chien H-YS, Honey CJ. 2020. Constructing and forgetting temporal context in the human cerebral cortex. *Neuron*

Dominey PF. 1998. A shared system for learning serial and temporal structure of sensori-motor sequences? Evidence from simulation and human experiments. *Brain Res Cogn Brain Res* 6: 163-72

Enel P, Procyk E, Quilodran R, Dominey P. 2016. Reservoir Computing Properties of Neural Dynamics in Prefrontal Cortex. *PLoS computational biology* 12

Hasson U, Yang E, Vallines I, Heeger DJ, Rubin N. 2008. A hierarchy of temporal receptive windows in human cortex. *Journal of Neuroscience* 28: 2539-50

Maass W, Natschlager T, Markram H. 2002. Real-time computing without stable states: a new framework for neural computation based on perturbations. *Neural Comput* 14: 2531-60

Rigotti M, Barak O, Warden MR, Wang XJ, Daw ND, et al. 2013. The importance of mixed selectivity in complex cognitive tasks. *Nature* 497: 585-90

Zadbood A, Chen J, Leong YC, Norman KA, Hasson U. 2017. How we transmit memories to other brains: constructing shared neural representations via communication. *Cerebral cortex* 27: 4988-5000
