# Supplementary material for: Narrative event segmentation in the cortical reservoir
Source: PLoS Comput Biol. 2021 Oct 7;17(10):e1008993. doi: 10.1371/journal.pcbi.1008993 (PMC8525778; doi:10.1371/journal.pcbi.1008993)
Supplement: Attachment — Submitted filename: R2-responses-current.docx [file pcbi.1008993.s002.docx]

Dear Editors Drs Theunissen, Gershman, and reveiwers Drs Nastase and Honey,

Thanks again for your editorial and review work. The questions and comments allowed for improvement of the manuscript. In this revision I have addressed all the comments, including the fast/slow asymmetry in the linear integrator.

Best regards,

P.F. Dominey

Reviewer comments, and responses:

***********************

Reviewer #1:

R1.1 In comparing the reservoir model to fMRI data, the author switched to a different story (the widely-used Sherlock movie and recall dataset). I’m a little curious why—but I assume it was just a matter of convenience.

* Yes – it was a matter of convenience, related to the reviewer’s suggestion to use data from segmented ROIs rather than whole brain data. In response to this I wanted to use fMRI data that had been validated in previous publications. I could not find ROI extracted data for Not The Fall, and so I used data from the angular gyrus from the Sherlock dataset. The source of the data is described in the manuscript.

R1.2 I’m curious about the temporal structure inherent in the reservoir network vs. the temporal structure inherent in the stimulus. For example, it could be interesting to scramble the word order of the stimulus prior to supplying it to the reservoir network (following Lerner et al., 2011).

* This is an interesting point. I am currently looking in more detail at the temporal response windows evaluated by scrambling the stimuli at word, sentence and paragraph levels as in Lerner et al. 2011. However, I consider presenting a meaningful analysis of effects revealed by scrambling would be a small studying in itself, and would blur the focus of the paper, and so I prefer to not go into that.

I now address this in the discussion page 19.:

“Lerner et al (2011) characterized a hierarchy of temporal response windows by examining inter-subject correlations in brain responses to audio that was intact, or scrambled at word, sentence or paragraph levels. It will be interesting to test the prediction that the virtual areas in the reservoir with their different alignment times will also display corresponding temporal response windows as revealed by scrambling at different levels of granularity as observed by Lerner et al (2011).”

R1.3 - Author summary: “brains are lead through” > “brains are led through”

* Corrected

R1.4 - Page 6: the sentence structure “and for developing the reservoir model” seems awkward

* Corrected

R1.5 - Page 10: “with p values ranging from p = 1.87e-10 for linear integrator 1 to p = 3.22e-02 for NIR 2”—it might be worth unpacking these results a bit more (or comparing models)

* The p values are now specified for all cases.

R1.6 - Page 10: “This means that the HMM can be used to measure similarity in event structure in neural activity from different stimulus modalities”—what exactly does this mean? We can run HMMs on two related datasets and then examine the event “templates” (i.e. centroids) found by the model across two datasets (but I don’t think that’s done here). Or we can compare the event boundaries, but this requires somehow matching the timing across datasets (e.g. in Chen et al., 2017, they use averaging to match events across perception and recall).

* Thanks. The point of this sentence was to indicate that previous research has demonstrated that there should be correspondence between event segmentation for the same narrative presented in different modalities. I have reformulated this sentence as: The HMM thus revealed significant correspondences between event segmentation of fMRI activity for movie-watching subjects and audio-narration subjects exposed to the same story with different modalities (movie vs. audio) and different timing.

Reviewer #2: Thanks to the author for a raft of new analyses and figures, and for actively engaging with all the points raised in the revision.

My only outstanding concerns relate to the Construction/Forgetting analyses, and to some conceptual and methodological points around that.

(Point 1) I think that the text could be a little more precise about what was shown empirically in relation to construction and forgetting, versus the underlying mechanisms. In Chien & Honey (2020), the finding was not precisely that construction was slow while forgetting was fast. Rather, the finding was that the alignment times (“time for construction of shared context”) increased from peripheral regions toward higher-order regions, while separation times (“forgetting”) did not increase in this systematic manner. It is true that, in the HAT model presented in that paper, there is an “event reset” that leads to rapid forgetting of prior context (leading to asymmetric behavior within the same model for construction and forgetting). However, the empirical (fMRI) finding from that study was not as definitive: the finding was just that the forgetting and construction patterns did not covary with each other: slow-constructing regions were not necessarily slow-forgetting regions. This absence of covariation observed in the empirical data is a kind of asymmetry, but it is not quite the same as saying that, for a single model of a single population, that the circuit can “construct slowly” and “forget quickly”. I think that the temporal asymmetry in individual models of individual regions is still an important point worth investigating — and there are plenty of other theoretical reasons to believe that some kind of context-resetting is occurring — but it is also important to be clear that the empirical finding (in relation to constructing-vs-forgetting timescales) was the absence of covariation across regions.

* Thanks for this clarification. I now make it clear for the empirical data from Chien and Honey that the point is that the forgetting and construction did not covary with each other. I also make this point with the model, and update Figure 9 to indicate how the time course for forgetting is uniform and for construction is distributed. I still use the word asymmetry, but when referring to the data I indicate that the assymetery is that there is a lack of covariance between construction and forgetting, rather that the fast/slow distinction.

(Point 2) I am having difficulty understanding how the linear integrator is showing a gradual ramping (for Different —> Same) and yet a rapid decrease (for Same —> Different). I am referring here to the top panel of Figure 5, and the following text in the revised manuscript: “Interestingly the same effects of abrupt forgetting and more progressive construction are observed for the linear integrator. This indicates that this asymmetry in constructing and forgetting is a property of leaky integrators systems.” In the “Integrator Details” section below I elaborate on why I think that linear integrators that are “slow to construct” should also be “slow to forget”; but similar arguments are presented in the Supplemental Material of Chien & Honey (2020).

* Yes, this is an unusual effect. I now analyse and explain how this effect is due to two factors. First, the signal in Figure 5 is not the activity of a single integrator, but rather it is the difference between two integrators. Second, in the same-different-same experiment, differences in the two integrators at the same-different transition are dominated by the differences in the inputs. At the different-same transition, differences are dominated by the memory, and subject to the leak-rate. Higher leak rates, produce the fading effect and emphasize the asymmetry. This is explained in a new paragraph near Figure 5 on p. 13, and then in more detail in the methods section, including a new Figure 14 that illustrates the effect of the leak rate on the asymmetry, near p. 25.

I am not sure where the discrepancy arises between the linear integrator used in this manuscript and the one used by Chien & Honey (2020). One possibility is that the integrator used here employs a different equation, although this is somewhat ambiguous (see Point 3 below).

* I have addressed this and now use a simplified integrator. This does not change the results – see below.

A second possibility is that the construction/forgetting effects interact in a crucial way with the autocorrelation in the input signals — the simulations in the current manuscript employ correlated input from the word embeddings, while the theoretical arguments of Chien & Honey (2020) made use of uncorrelated input signals. To test for the role of autocorrelated input, one possibility would be to re-run the construction / forgetting analysis on “fake stimuli” constructed as random and independent sequences of vectors generated by sample Gaussian-distributed random numbers. If the asymmetry goes away, then it seems that the sequential properties of the stimulus play a role.

* We performed this experiment, using uncorrelated input, and the effect remains. This is explained in a new paragraph in the methods section on the linear integrator.

A third possibility is that the dependent variable [“difference in activation level”] used in the Figure 5 analyses is different in some important way from the dependent variable (pattern correlation) used in Chien & Honey (2020). However, this possibility seems unlikely, as the current manuscript also shows similar asymmetry effects using a pattern correlation measure.

* We investigated this – the effect is not due to some difference in the pattern correlation measure, but rather to the effects of time constant of the linear integrator in the context of this task, as now explained.

Related to the difference between these results and those of Chien and Honey (2020), I also note the following “In the linear integrator of Chien and Honey (2020), it is likely that with relatively high leak rates (i.e. low vales of parameter ρ*i* < 0.5 in their Equation 1 describing the linear integrator), this asymmetry would not be present.” At the end of the methods section on the asymmetry, near p. 26.

(Point 3) The equations describing the leaky integrator seems to contain a typo, so it is not totally clear what form of integration was employed. In the text, the equation reads as:

LI(n+1) = ( (1-alpha)LI(n-1)n + (1+alpha)*embedding(n) )/(n+1)

where I believe there may be an erroneous multiplication of LI by n?

* Good point. We initially implemented an integrator that would weigh the current input by 1/n, and we thus multiplied the memory by n and divided the whole sum by n+1 as initially written. Finally, we observe that this has an excessive fading memory effect, and that it is more complicated than the standard form of linear integrator, as noted by the reviewer. We thus simplify the linear integrator equation as suggested to the following.

LI(n) = (1-alpha)*LI(n-1) + (1+alpha)*embedding(n)

We reran all experiments with the integrator using this simplified equation. The results are unchanged.


(Point 4) Assuming that a key conceptual contribution of the present manuscript is that the reservoir computing model has a repertoire of multiple time constants, then — to be clear — is the neural reservoir being proposed as a model of multiple stages of cortical processing (each with their own timescale)? This is important because one could also imagine the reservoir as a model of a single region, in which distinct nodes within that region each have their own timescale (as in the work of Bernacchia et al, for example). Please clarify in the text.

* We now clarify that this effect should be expressed both at a local level – with a diversity of time constants within an area, and across multiple areas, with a hierarchy of time constants as one moves farther from the input. This is explained in the discussion near page 20.
~~~~
Integrator Details
~~~~

Can linear integrators be “slow constructors” and “rapid forgetters”? Informally, I want to say something like “integrators that are slow to forget prior context, should also be slow to absorb new input”.

* Thanks for this analysis. I have now provided details showing that the slow constructor and rapid forgetter asymmetry that we observe arises when we look at the difference between two integrators, with a sufficiently slow leak rate. Informally, forgetting is dominated by input, largely independent of the leak rate, and thus occurs rapidly. Constructing is dominated by the memory term, and can thus be arbitrarily slow, depending on the leak rate.

This is now addressed in the extended section on the integrator in the methods section near page 25.
